# MOF-Derived CeO_2_ Nanorod as a Separator Coating Enabling Enhanced Performance for Lithium–Sulfur Batteries

**DOI:** 10.3390/molecules29081852

**Published:** 2024-04-18

**Authors:** Hao Xiao, Jian Qin, Haodong Wang, Xiaoxu Lai, Pei Shi, Chi Chen, Dan Sun

**Affiliations:** 1 College of Chemistry, Fuzhou University, Fuzhou 350116, China; xmxiaohao@fjirsm.ac.cn (H.X.); xmqinjian@fjirsm.ac.cn (J.Q.); 2 CAS Key Laboratory of Design and Assembly of Functional Nanostructures, and Fujian Provincial Key Laboratory of Nanomaterials, Fujian Institute of Research on the Structure of Matter, Chinese Academy of Sciences, Fuzhou 350002, China; whd19855695910@163.com (H.W.); xmlaixiaoxu@fjirsm.ac.cn (X.L.); 348936@whut.edu.cn (P.S.); 3 Xiamen Institute of Rare Earth Materials, Haixi Institutes, Chinese Academy of Sciences, Xiamen 361021, China; 4 Xiamen Key Laboratory of Rare Earth Photoelectric Functional Materials, Xiamen 361021, China; 5 Fujian College, University of Chinese Academy Sciences, Fuzhou 350002, China

**Keywords:** lithium–sulfur batteries, metal organic frameworks, cerium oxides, modified separator, synergistic effect

## Abstract

The deployment of Li–S batteries in the commercial sector faces obstacles due to their low electrical conductivity, slow redox reactions, quick fading of capacity, and reduced coulombic efficiency. These issues stem from the “shuttle effect” associated with lithium polysulfides (LiPSs). In this work, a haystack-like CeO_2_ derived from a cerium-based metal-organic framework (Ce-MOF) is obtained for the modification of a polypropylene separator. The carbon framework and CeO_2_ coexist in this haystack-like structure and contribute to a synergistic effect on the restriction of LiPSs shuttling. The carbon network enhances electron transfer in the conversion of LiPSs, improving the rate performance of the battery. Moreover, CeO_2_ enhances the redox kinetics of LiPSs, effectively reducing the “shuttle effect” in Li–S batteries. The Li–S battery with the optimized CeO_2_ modified separator shows an initial discharge capacity of 870.7 mAh/g at 2 C, maintaining excellent capacity over 500 cycles. This research offers insights into designing functional separators to mitigate the “shuttle effect” in Li–S batteries.

## 1. Introduction

Boasting a high theoretical capacity (1675 mAh/g), high energy density (2600 Wh/kg), abundant availability (260 ppm in the Earth’s crust), low cost, and environmental benefits, lithium–sulfur (Li–S) batteries are seen as promising candidates for future energy storage systems [1,2,3,4]. Nevertheless, the commercial deployment of Li–S batteries faces challenges primarily due to the “shuttle effect” [5]. This phenomenon arises as soluble polysulfides (Li_2_S_x_, 4 ≤ x ≤ 8) migrate from the sulfur cathode to the lithium metal anode during charge and discharge cycles due to concentration gradients and electric fields, leading to significant irreversible capacity loss and a diminished cycle life [6,7,8]. Additionally, the poor electrical conductivity of elemental sulfur and Li_2_S/Li_2_S_2_ results in low sulfur utilization and suboptimal rate performance, further constraining the potential applications of Li–S batteries. To tackle these challenges, numerous researchers have dedicated their efforts toward the development and implementation of modified separators [1].

Separator-modified materials are classified into nonpolar and polar materials, such as carbon (porous carbon spheres [9], carbon nanotubes [10], and graphene [11,12], etc.) and transition metal compounds (metal oxides [13], metal carbides [14], metal nitrides [15], and metal sulfides [16], etc.), respectively. Although carbon materials are highly conductive and enable efficient charge transfer during the conversion of LiPSs, polar materials are more widely used to suppress the “shuttle effect” due to their strong affinity to polar LiPSs.

Rare earth (RE) elements differ from transition metal elements due to their distinctive 4f shell electronic configurations, which result in unique physical and chemical properties. Among rare earth materials, rare-earth oxides (REOs) have garnered significant attention due to their applications in optics, electronics, magnetism, catalysis, and energy conversion [17,18,19]. In particular, CeO_2_, a representative REOs, has attracted interest in catalyzing the conversion of LiPSs. This is because CeO_2_ possesses exceptional catalytic activity, which arises from the reversible Ce^4+^/Ce^3+^ redox couple facilitated by surface oxygen vacancies [20]. This redox couple serves as an efficient active site that accelerates the conversion and decomposition of LiPSs at the interface. Recent research on REOs has focused on achieving nanometer-scale dimension to enhance the conversion of LiPSs. However, traditional synthesis methods for cerium oxide hard to avoid particle agglomeration, posing challenges in obtaining high-activity REOs. In light of this, it is worthwhile to explore alternative synthesis methods to obtain REOs with improved activity.

Metal-organic frameworks (MOFs) are typically composed of metal nodes and organic linkers, resulting in materials that are porous and possess tunable pore sizes and relatively large specific surface areas [21,22]. The unique frame structure of MOFs enables the use of MOFs as sacrifice templates, resulting in superior performance compared to conventional synthesis methods of REOs. The framework of MOFs enables the even dispersion of MOFs-derived oxides on its network, preventing the agglomeration of nano oxides. Furthermore, the large specific surface areas of MOFs promote the exposure of active sites of oxides. Additionally, the framework of MOFs facilitates channels for electron and ion transport, thereby accelerating the conversion of LiPSs [23,24,25,26].

Drawing inspiration from the aforementioned factors, we developed a novel CeO_2_ material derived from MOFs, showcasing a haystack-like structure through the use of MOFs as sacrificial templates. This CeO_2_ material was utilized for enhancing the separators within Li–S batteries. The incorporation of a carbon network derived from the organic framework of Ce-MOF aids in promoting electron transfer during the conversion of LiPSs, thereby enhancing the rate capability of the Li–S batteries. Additionally, the CeO_2_ exhibits remarkable electrocatalytic activity, facilitating the acceleration of redox reactions of LiPSs and effectively mitigating the shuttle effect within the batteries. The Li–S battery assembled with a CeO_2_ modified separator exhibits a high specific capacity of 1260 mAh/g at a rate of 0.2 C, an excellent rate performance of 662.8 mAh/g at a rate of 5 C, and a capacity decay of only 0.1% per cycle after 500 cycles at a rate of 2 C, demonstrating significant cycling stability.

## 2. Results and Discussion

**Scheme 1:**
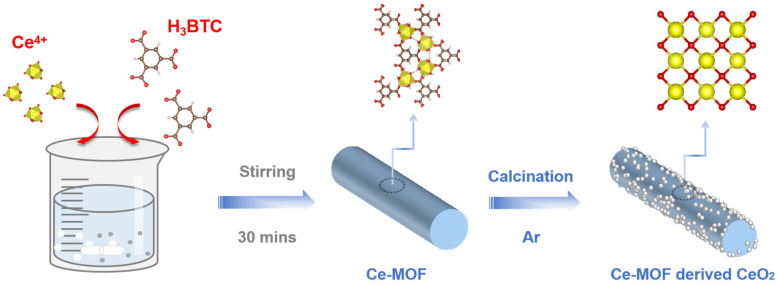
Schematic illustration of the Ce-MOF and CeO_2_ synthesis.

The progresses of Ce-MOF and CeO_2_ synthesis are illustrated in Scheme 1. The Ce-MOF is prepared by a hydrothermal method with Ce(NO_3_)_3_·6H_2_O and 1,3,5-Benzenetricarboxylic acid as raw materials. To obtain the CeO_2_, the Ce-MOF is used as precursor calcinated at 800 °C under an argon atmosphere. Subsequently, the CeO_2_ separator is produced by a common Li–S batteries slurry coating method.

**Figure 1:**
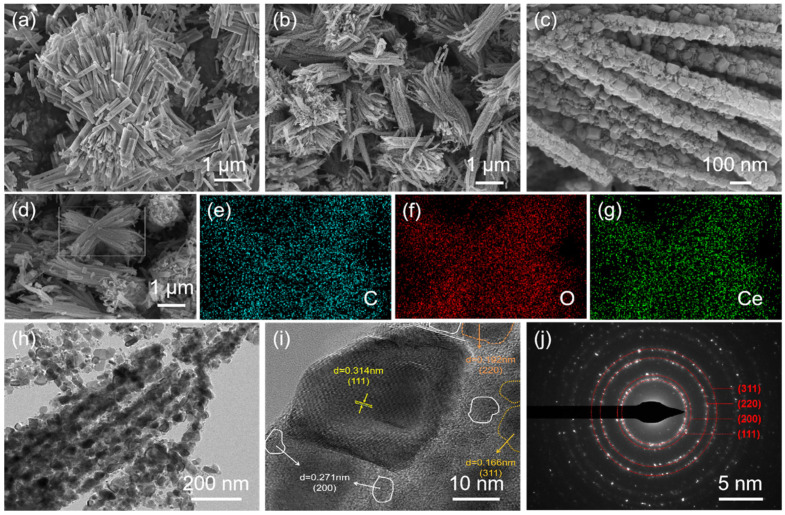
(**a**) SEM image of Ce-MOF; (**b**,**c**) SEM images of CeO_2_; (**d**) SEM image of selected area for CeO_2_; (**e**–**g**) corresponding elemental mappings of C, O, Ce, respectively; (**h**) TEM image, (**i**) HRTEM image, and (**j**) SAED pattern of CeO_2_.

Transmission electron microscopy (TEM) and scanning electron microscopy (SEM) analyses are performed to reveal the appearance differences between Ce-MOF and CeO_2_, as depicted in Figure 1. The SEM images (Figure 1a,b) demonstrate that CeO_2_ retains a haystack-like morphology reminiscent of the Ce-MOF precursor, indicating that the calcined CeO_2_ maintains the structural characteristics of Ce-MOF. Notably, the haystack-like CeO_2_ is composed of nanorods with an average diameter ranging from 40 to 80 nm, as depicted in Figure 1c. Furthermore, the uniform distribution of carbon, oxygen, and cerium elements depicted in the SEM image (Figure 1d) and the corresponding elemental mappings (Figure 1e–g) confirms the retention of the carbon component in Ce-MOF-derived CeO_2_. The morphological features and size of CeO_2_ observed in the TEM image (Figure 1h) correspond with the findings from the SEM images. Furthermore, the high-resolution TEM (HRTEM) imagery (seen in Figure 1i) shows lattice fringe spacings of 0.314, 0.271, 0.192, and 0.166 nm, aligning with the (111), (200), (220), and (311) planes of CeO_2_, respectively [27,28]. The selected area electron diffraction (SAED) pattern in Figure 1j illustrates the (111), (200), (220), and (311) facets of CeO_2_, further corroborating the successful synthesis of CeO_2_ derived from Ce-MOF.

**Figure 2:**
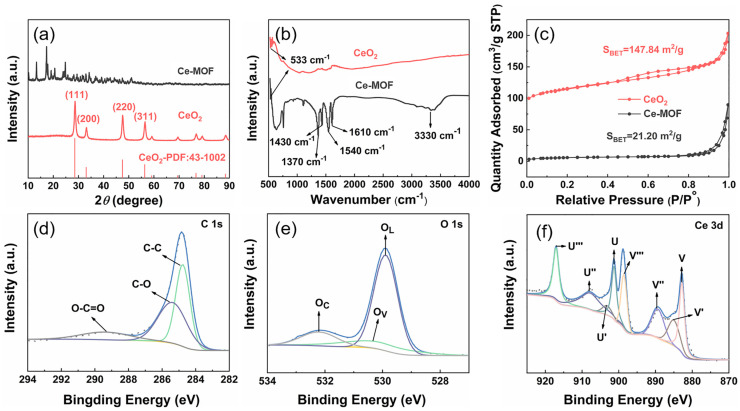
(**a**) XRD patterns, (**b**) FTIR patterns, and (**c**) N_2_ adsorption-desorption isotherms for Ce-MOF and CeO_2_; (**d**–**f**) XPS spectra of C 1s, O 1s, Ce 3d for CeO_2_.

Figure 2a displays the X-ray diffraction (XRD) patterns of Ce-MOF and CeO_2_. The diffraction peaks of Ce-MOF align closely with previous research [27] and the peaks of the synthesized CeO_2_ can be indexed well with CeO_2_ (JCPDS: 43-1002). To study the bonding features of Ce-MOF and CeO_2_, FT-IR spectroscopy was performed and the results are shown in Figure 2b. For Ce-MOF, two peaks at around 1610 cm^−1^ and 1540 cm^−1^ can be attributed to stretching vibrations of asymmetric carboxylate, while the peaks at around 1430 cm^−1^ and 1370 cm^−1^ are assigned to symmetric carboxylate anions. The peak at 3330 cm^−1^ belongs to water (O–H). After calcination, the peak located at 533 cm^−1^ in the spectrum of CeO_2_ is classified as to the Ce–O vibration that is in accord with the Ce-MOF. The specific surface areas of Ce-MOF and CeO_2_ are explored using Brunauer–Emmett–Teller (BET) analysis through N_2_ adsorption–desorption isotherms, as presented in Figure 2c. Compared to the Ce-MOF, the specific surface areas of CeO_2_ are increased from 21.20 m^2^/g to 147.84 m^2^/g, leading to stronger adsorption ability to LiPSs. The pore size distributions in Figure S2 reveal that CeO_2_ has a porous structure.

The chemical states of the elements present on the CeO_2_ surface are examined using X-ray photoelectron spectroscopy (XPS). Figure 2d shows three peaks in the C 1s spectrum, which is assigned to C–C, C–O, and O–C=O (284.7, 285.4, and 289.3 eV), respectively [19]. The O 1s spectrum (Figure 2e) displays three peaks at 529.9, 530.3, and 532.2 eV, which could be indexed to lattice oxygen (O_L_), O^2−^ ions in surface oxygen vacancies (O_V_), and chemisorbed oxygen species (O_C_), respectively [29]. As depicted in Figure 2f, the high-resolution Ce 3d spectrum is fitted into eight peaks as follows: V (882.8 eV), V′ (885.2 eV), V″ (889.6 eV), V‴ (898.5 eV), U (901.2 eV), U′ (902.9 eV), U″ (908.1 eV), U‴ (917.2 eV) [29,30]. The binding energies of the peaks V/U, V″/U″, and V‴/U‴ correspond to the standard spectra of Ce^4+^, while the binding energies of V′ and U′ correspond to Ce^3+^. This demonstrates the presence of Ce^4+^/Ce^3+^ redox couples in the CeO_2_ material.

**Figure 3:**
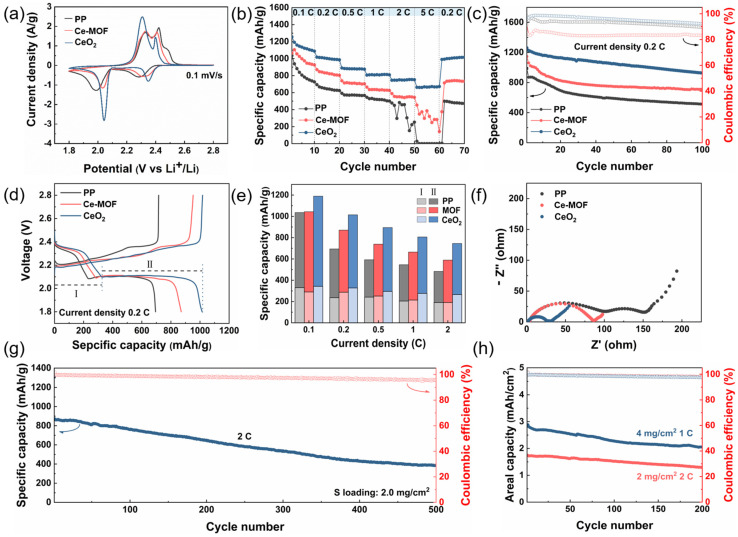
(**a**) CV curves of Li–S batteries with PP, Ce-MOF, and CeO_2_ modified separators at a scan rate of 0.1 mV/s, (**b**) Cycling performances at 0.2 C and (**c**) rate performances of Li–S batteries with PP, Ce-MOF, and CeO_2_ modified separators; (**d**) charge–discharge profiles of cells with PP, Ce-MOF, and CeO_2_ modified separators at 0.2 C; (**e**) discharge capacity of cells with PP, Ce-MOF, and CeO_2_ modified separators for plateau I and plateau II at various current densities; (**f**) EIS spectra of cells with PP, Ce-MOF, and CeO_2_ modified separators; (**g**) long-term performance of the Li–S battery with a CeO_2_ modified separator at a current density of 2 C for 500 cycles; (**h**) the areal capacity of the Li–S battery with high sulfur loading at the current density of 2 C and 1 C, respectively.

The SEM images displayed in Figure S3 reveal the even distribution of Ce-MOF and CeO_2_ on the PP separator in a 3D structure, which functions as a physical barrier against LiPSs. The cyclic voltammetry (CV) curves provided in Figure 3a show the performance of Li–S batteries utilizing PP, Ce-MOF, and CeO_2_ modified separators under a scan rate of 0.1 mV/s within a voltage window of 1.8 to 2.8 V (vs. Li^+^/Li). The reduction peaks at 2.3 and 2.05 V signify the reduction of sulfur (S_8_) to long-chain soluble LiPSs (Li_2_S_x_, where 4 ≤ x ≤ 8), transitioning into short-chain insoluble Li_2_S_2_/Li_2_S for both Ce-MOF and CeO_2_ [31,32]. On the contrary, the oxidation peaks at approximately 2.3 and 2.4 V correspond to the reverse process of Li_2_S/Li_2_S_2_ conversion back to S_8_ [33]. Notably, batteries with CeO_2_ modified separators demonstrate higher peak currents, reaching around 2 A/g, and a smaller polarization. These outcomes suggest that the incorporation of CeO_2_ enhances the redox reactions of polysulfides and diminishes polarization.

Figure 3b depicts the rate performance of cells with PP and modified separators across current densities from 0.1 to 5 C. The cell equipped with a CeO_2_ modified separator exhibits remarkable specific capacities of 1256.2, 1017.7, 890.1, 806.4, 745.9, and 662.8 mAh/g at 0.1, 0.2, 0.5, 1, 2, and 5 C, respectively, outperforming cells with PP and Ce-MOF modified separators. Conversely, the PP and MOF separators demonstrate poor stability at current densities of 2 C and 5 C. Interestingly, upon reducing the current density from 5 C to 0.2 C, the cell with a CeO_2_ modified separator displays outstanding reversibility and stability, rebounding to 990.9 mAh/g, equivalent to approximately 97.4% of the initial value. Furthermore, Figure 3c illustrates the cycling performances of cells utilizing various separators at a current density of 0.2 C. Notably, the cell featuring a CeO_2_ modified separator showcases superior cycling capability, initiating with a specific capacity of 1260 mAh/g and the highest coulombic efficiency. Even after 100 cycles, the specific capacity of the cell with a CeO_2_ modified separator remains at 926.9 mAh/g, significantly exceeding those with Ce-MOF modified (709.2 mAh/g) and PP (513.2 mAh/g) separators.

The galvanostatic discharge/charge (GDC) profiles for cells incorporating various separators at current densities from 0.1 C to 2 C are detailed in Figure 3d and Figure S4. Notably, Figure 3d shows two distinct discharge plateaus at a current density of 0.2 C. The initial plateau relates to the conversion of S_8_ to long-chain soluble LiPSs (Li_2_S_x_, 4 ≤ x ≤ 8) (I), followed by the conversion of these long-chain LiPSs to short-chain Li_2_S_2_/Li_2_S (II). Subsequently, Figure 3e and Table S1 present a summary of the specific capacities associated with plateaus I and II and the ratio between plateau II and the discharge capacity at current densities ranging from 0.1 C to 2 C. Notably, despite capacity fade with increasing current rate due to an insufficient reduction of LiPSs, cells with PP, Ce-MOF, and CeO_2_ separators exhibit around 60.5%, 67.6%, and 64.4% of the discharge capacity corresponding to plateau II at a current density of 2 C, respectively. This observation suggests that the modified separators facilitate the conversion of long-chain Li_2_S_4_ to Li_2_S_2_/Li_2_S [34]. Furthermore, the electrochemical impedance spectra (EIS) of cells with different separators are depicted in Figure 3f. Of particular interest is the cell with a CeO_2_ modified separator, which demonstrates the lowest charge transfer resistance (*R_ct_*). This outcome indicates the enhanced charge transfer kinetics of CeO_2_, potentially attributed to the carbon component within CeO_2_ derived from Ce-MOF.

For Li–S batteries to reach commercialization and large-scale application, parameters like long-term cycling stability and high areal capacity are essential. Demonstrated in Figure 3g, the cell employing a CeO_2_ modified separator showed remarkable long-term cycling stability during tests at a current density of 2 C with a sulfur loading of 2 mg/cm^2^. Initially, it delivered a specific capacity of 870.7 mAh/g at 2 C and experienced a capacity decay of only 0.1% per cycle after 500 cycles, all while maintaining a stable coulombic efficiency exceeding 95%. In addition, to investigate the high areal capacity of cell with a CeO_2_ modified separator, the areal sulfur mass loading is increased to 4 mg/cm^2^ (Figure 3h). High initial areal capacity of 1.75 and 2.89 mAh/cm^2^ are achieved at the current density of 2 C and 1 C, and retained the areal capacities of 1.30 and 2.05 mAh/cm^2^ after 200 cycles, demonstrating that the CeO_2_ modified separator possesses the potential in Li–S batteries applications.

**Figure 4:**
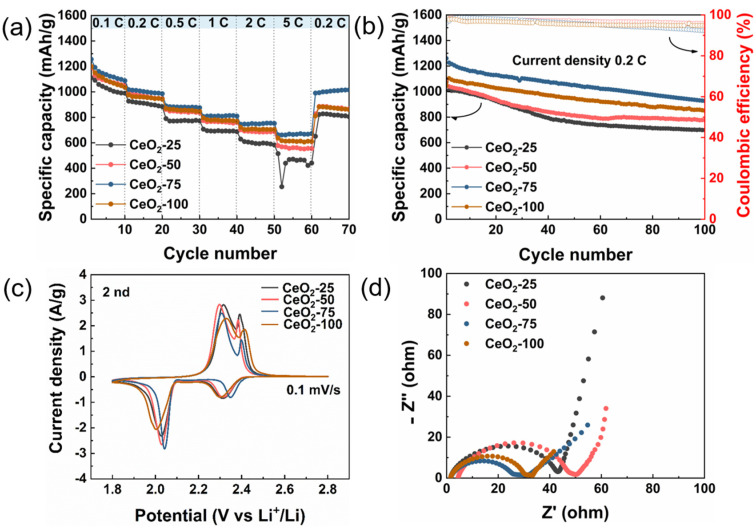
Electrochemical performances of CeO_2_-25, CeO_2_-50, CeO_2_-75, and CeO_2_-100. (**a**) Rate performances at various current density of Li–S batteries with various thicknesses; (**b**) cycling performances of Li–S batteries with various thicknesses at 0.2 C; (**c**) CV curves of Li–S batteries with various thicknesses at a scan rate of 0.1 mV/s; (**d**) EIS spectra of Li–S batteries with various thicknesses.

To investigate the influence of CeO_2_ modified separator thickness on the performance of Li–S batteries, cells with varying thicknesses of CeO_2_ modified separators (referred to as CeO_2_-25, 50, 75, and 100) were prepared using a doctor’s blade. The actual coating thicknesses of CeO_2_-25, CeO_2_-50, CeO_2_-75, and CeO_2_-100 were measured as 12.84, 14.33, 16.19, and 18.42 μm, respectively, as shown in Figure S5 and Table S2. The rate performances of cells with different thicknesses of CeO_2_ modified separators are illustrated in Figure 4a, highlighting that the cell with the CeO_2_-75 modified separator exhibited the highest specific capacity of 1256.2 mAh/g at 0.1 C and 662.8 mAh/g at 5 C. Upon reducing the current density to 0.2 C, the specific capacity of the cell with the CeO_2_-75 modified separator increased to 990.9 mAh/g, surpassing those of cells with CeO_2_-25, 50, and 100.

In addition, the cycling performances of the cell with CeO_2_ modified separators with different thicknesses are investigated in Figure 4b. The maintained discharging capacity of the cell with the CeO_2_-75 modified separator after 100 cycles at current density 0.2 C is 926.9 mAh/g, much higher than those of cells with the CeO_2_-25 (698.8 mAh/g), CeO_2_-50 (776.6 mAh/g), and CeO_2_-100 (851.0 mAh/g) modified separator. As shown in Figure 4c, the CV curves of batteries using CeO_2_-75 modified separators demonstrate the highest current intensity, and among all samples, CeO_2_-75 has the highest reduction peak potential and the lowest oxidation peak potential, with a polarization value of about 250 mV, further indicating its fastest reaction kinetics. As depicted in Figure S6, the CV curves from the first to fifth cycles of the cell with CeO_2_-75 at a scan rate of 0.1 mV/s are measured, showing that there is no significant change in the potential of redox peaks, thereby suggesting superior electrochemical stability [35].

As illustrated in Figure 4d, the electrochemical impedance spectra of cells with various thicknesses of CeO_2_ modified separators are investigated. Among them, the cell with the CeO_2_-75 modified separator shows the smallest *R_ct_*, revealing the fast kinetics of LiPSs redox. The value of *R_ct_* varies in a trend in which CeO_2_-75 > CeO_2_-100 > CeO_2_-50 > CeO_2_-25. Based on all electrochemical results of cells with various thicknesses of CeO_2_ modified separators, we found that increasing the thicknesses of CeO_2_ coatings can restrain the “shuttle effect” and accelerate the conversion of LiPSs to some extent. However, when the coating is too thick, the transmission of electrons and ions will be inhibited, leading to worse electrochemical performances. Therefore, the thicknesses of modified separators would be worth further attention in future research.

**Figure 5:**
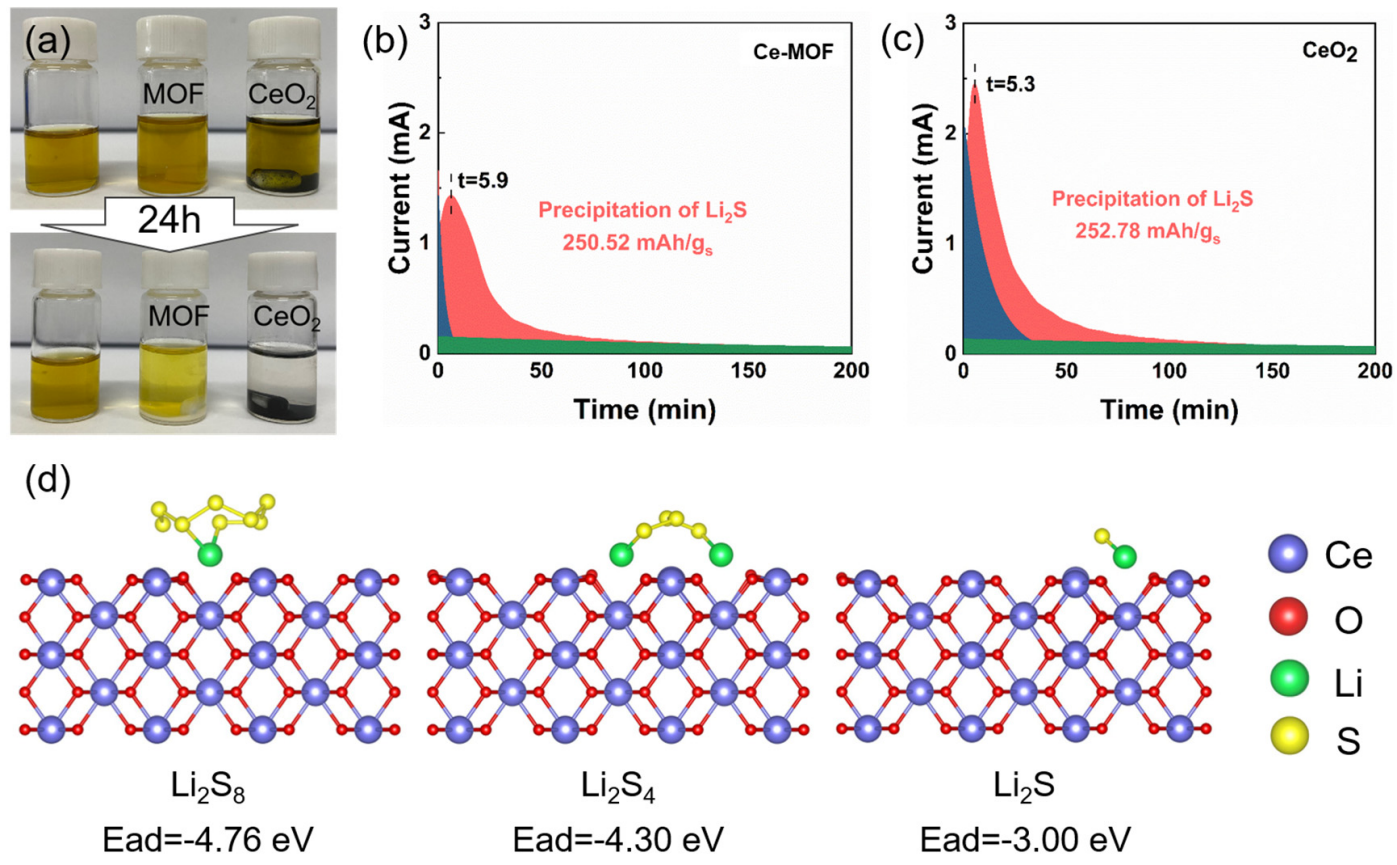
(**a**) Digital images of the polysulfide (Li_2_S_9_) adsorption test of Ce-MOF and CeO_2_; potentiostatic discharge profiles of Li_2_S_8_ solution on (**b**) Ce-MOF and (**c**) CeO_2_ at 2.05 V, the peak areas in red, blue, and green represent the precipitation of Li_2_S, the reduction of Li_2_S_8_, and the reduction of Li_2_S_6_, respectively; (**d**) adsorption energy for Li_2_S_8_, Li_2_S_4,_ and Li_2_S on CeO_2_. Herein, purple, red, green, and yellow balls represent cerium, oxygen, lithium, and sulfur, respectively.

As shown in Figure 5a, the polysulfide adsorption experiment is conducted to verify the chemical binding ability of Ce-MOF and CeO_2_ [36]. Samples of 10 mg were added into 3 mL of 5 mM Li_2_S_9_ solution. The Li_2_S_9_ solution with CeO_2_ become complete colorless while the Li_2_S_9_ solution with Ce-MOF is light yellow after 24 h, demonstrating that CeO_2_ exhibits much stronger chemical interactions with LiPSs than Ce-MOF. The formation mechanism of Li_2_S is investigated by Li_2_S nucleation experiments as shown in Figure 5b,c. The peak areas in red, blue, and green represent the precipitation of Li_2_S, the reduction of Li_2_S_8_, and the reduction of Li_2_S_6_, respectively. Compared to the cell with the Ce-MOF modified separator, a sharp nucleation peak for the cell with the CeO_2_ modified separator appears earlier, demonstrating a faster nucleation ability of the Li_2_S [37,38]. The capacities of the precipitated Li_2_S on the cell with Ce-MOF and CeO_2_ modified separators are calculated as 250.52 and 252.78 mAh/g, respectively.

Furthermore, density functional theory (DFT) simulations are conducted to certify the chemical interaction between CeO_2_ and LiPSs. Figure 5d infers that the intermediate Li_2_S_8_, Li_2_S_4_, and Li_2_S are chosen as the computational models to calculate the adsorption energy. The relaxed configurations of Li_2_S_8_, Li_2_S_4_, and Li_2_S adsorbed on the surface of CeO_2_ deliver the binding energies of −4.76, −4.30, and −3.00 eV, respectively. These values are comparable or superior to other materials as reported elsewhere [7,9], indicating a strong chemical adsorption. Additionally, compared to Li_2_S, CeO_2_ has lower binding energy to the long-chain LiPSs, demonstrating the stronger the polar–polar interactions between CeO_2_ and LiPSs [39]. Overall, the DFT calculation results suggest that the CeO_2_ can effectively capture LiPSs.

## 3. Materials and Methods

### 3.1. Synthesis of the MOF

The Ce-MOF was synthesized by a conventional hydrothermal method as reported elsewhere [27]. A solution of Ce(NO_3_)_3_·6H_2_O at 0.5 M concentration was initially prepared. A mixture of deionized water and ethanol (99%) was used as the solvent, with PVP (10 wt%) and 0.5 mmol of 1,3,5-Benzenetricarboxylic acid dissolved in it. The Ce(NO_3_)_3_·6H_2_O solution was then gradually mixed into this solvent under vigorous stirring at ambient temperature for 30 min. After stirring, the mixture underwent filtration and was washed with deionized water and ethanol to obtain the Ce-MOF powder.

### 3.2. Synthesis of the CeO_2_

The CeO_2_ powder was synthesized through a high-temperature calcination process. The preobtained Ce-MOF powder was placed in a tube furnace and calcined at 800 °C for 5 h under an argon gas flow, with the heating rate set at 5 °C/min, as determined by the TG curve (Figure S1).

### 3.3. Synthesis of CeO_2_ Coating Separator

A mixture containing CeO_2_ powder, Super P, and PVDF in an 8:1:1 weight ratio was prepared as a slurry and coated onto a polypropylene (PP) separator using a doctor blade technique. This coated separator was then dried in a vacuum oven at 50 °C overnight. The resultant modified separator was cut into circles for cell assembly.

### 3.4. Sulfur Cathode Preparation

The sulfur mass loading of the cathodes is 1.0–1.5 mg/cm^2^. The sulfur cathodes were crafted by spreading a slurry mixture of sulfur powder, Super P, CMC, and SBR (55:35:5:5 weight ratio) onto an aluminum substrate. This mixture was dried in a vacuum oven at 80 °C for 12 h, resulting in cathodes with a sulfur mass loading between 1.0–1.5 mg/cm^2^, ready for cell assembly.

### 3.5. Electrochemical Measurements

For assessing the electrochemical performance of the modified separators, type CR2025 coin cells were constructed with lithium metal serving as the anode. The electrolyte, freshly prepared with lithium bis((trifluoromethyl)sulfonyl) imide (1 M) in 1,3-dioxolane (DOL) and 1,2-dimethoxyethane (DME) containing LiNO_3_ (1 wt%) with a volume ratio of 1:1, was carefully manipulated in an argon-filled glove box. Subsequently, LAND battery systems were employed for galvanostatic charge/discharge testing and rate performance evaluation. Cyclic voltammetry (CV) curves within a 1.8–2.8 V window and electrochemical impedance spectroscopy (EIS) measurements were conducted at a 0.1 mV/s scan rate and a 10 mHz to 100 kHz frequency range on an electrochemical workstation (MULTI AUTOLAB M204).

### 3.6. Materials Characterization

To certify the structure of the obtained samples, X-ray powder diffraction (XRD, Miniflex 600, Rigaku, Tokyo, Japan) patterns were measured with the Cu-Kα radiation at a scan rate of 5°/min. The calcined temperature range, essential for this study, was determined using thermogravimetric analysis (TAG, TGA/DSC) in an Ar atmosphere, spanning from 30 to 1000 °C. Fourier-transformed infrared spectra (FT-IR, Nicolet iS 50, Thermo Scientific, Waltham, MA, USA) were then employed for a more comprehensive analysis of the chemical bonds. N_2_ adsorption isotherms (Autosorb-iQ, Anton Paar, Boynton Beach, FL, USA) were utilized to determine the Brunauer–Emmett–Teller (BET) surface area. The sample morphology was further analyzed using a field-emission scanning electron microscope (FESEM, Apreo S LoVac, Thermo Scientific, Waltham, MA, USA) at a 5.0 kV acceleration voltage, equipped with an Energy Dispersive Spectrometer (EDS), and a transmission electron microscope (TEM, FEI Talos F200s, FEI, Hillsboro, OR, USA). Finally, to gain insights into the chemical compositions, X-ray photoelectron spectroscopy (XPS, Axis Supra, Shimadzu, Tokyo, Japan) was conducted.

### 3.7. Polysulfides Adsorption Experiment

Li_2_S_9_ solution was prepared by sulfur reaction with Li_2_S (*n*:*n* = 8:1) in DOL/DME (1:1) under 50 °C overnight. Appropriate MOF and CeO_2_ powder were added into 3 mL Li_2_S_9_ solution. A blank group without any powder was measured. All processes are carried out in an argon-filled glove box.

### 3.8. Measurement of the Li_2_S Nucleation

In the cell configuration, two pieces of carbon cloth served as electrodes, which were isolated by a separator modified with either Ce-MOF or CeO_2_. Specifically, on the cathode, 20 μL of Li_2_S_8_ (2.5 mM) was introduced, whereas on the anode, 20 μL of blank electrolyte was dispensed. The cell underwent a galvanostatic discharge process at a current of 0.112 mA until reaching a voltage of 2.06 V. Subsequently, it was maintained at 2.05 V until the current dwindled to 10^−5^ A.

### 3.9. Computational Method

The first-principles computations in this study were conducted using density functional theory (DFT) within the Vienna Ab Initio Simulation Package (VASP 5.4) [40]. To ensure accurate results, the Perdew–Burke–Ernzerhof (PBE) parametrization of the generalized gradient approximation (GGA) and projector-augmented wave (PAW) were employed [41,42]. A 2 × 2 × 1 Γ-centered k-mesh was chosen to represent the Brillouin zone, with a plane-wave energy cutoff of 520 eV. Energy convergence criteria were set at less than 10^−5^ eV per atom, and force convergence calculations were set at 0.03 eV/Å. Furthermore, the van der Waals interaction (vdWs) between CeO_2_ and LiPSs was accurately evaluated through the use of the DFT-D3 method for geometry optimization and energy calculations [43]. A vacuum space of 20 Å was set to avoid the interaction between layers. The calculation of GGA+U was performed by using a model based on the previously proposed model, where the value of Ueff (Ueff = Coulomb U − exchange J) for Ce is 6.3 eV. Spin polarization was factored into all calculations. In addition, the structure drawing and charge density visualization were generated using VESTA 3.9 [44].

The binding energy (*E_ad_*) of LiPSs on the CeO_2_ surface was defined as

$$E_{ad}=E_{host+guest}-E_{host}-E_{guest}$$

where *E_host+guest_* represents the total energy of CeO_2_ with an adsorbed LiPSs molecule, *E_host_* is the total energy of the CeO_2_, and *E_guest_* is the total energy of the LiPSs.

## 4. Conclusions

This work successfully synthesized a Ce-MOF-derived CeO_2_ modified separator for the Li–S battery, which has a synergistic effect on the restriction of LiPSs migration. The carbon framework derived from Ce-MOF provides enhanced charge transfer dynamics and the polar CeO_2_ exhibits a strong chemical adsorption to LiPSs during the charging–discharging progress, effectively accelerating the conversion of LiPSs. In summary, the battery featuring a separator augmented with CeO_2_ derived from Ce-MOF delivers a remarkable specific capacity of 1260 mAh/g at a current density of 0.2 C, demonstrates outstanding rate performance of 662.8 mAh/g at 5 C, and a low capacity decay of 0.1% per cycle over 500 cycles at 2 C. This study introduces an innovative approach to designing functional separators that effectively mitigate the “shuttle effect”, significantly boosting the performance of Li–S batteries.

## Data Availability

Data are contained within the article and Supplementary Materials.

## Supplementary Materials

The following supporting information can be downloaded at: https://www.mdpi.com/article/10.3390/molecules29081852/s1, Figure S1: Thermogravimetric analysis curve of Ce-MOF to determine the calcination temperature for the synthesis of CeO_2_; Figure S2: Pore size distributions of Ce-MOF and CeO_2_, respectively; Figure S3: SEM images of (a,d) pristine polypropylene (PP) separator, (b,e) Ce-MOF modified separator and (c,f) CeO_2_ modified separator; Figure S4: Charge-discharge profiles of Li–S batteries with (a) PP separator, (b) Ce-MOF, and (c) CeO_2_ modified separators at different current densities of 0.1, 0.2, 0.5, 1, 2 C; Figure S5: Cross-sectional SEM images of (a) CeO_2_-25, (b) CeO_2_-50, (c) CeO_2_-75 and (d) CeO_2_-100 modified separators; Figure S6: The first, third and fifth cycled CV curves of Li–S batteries with CeO_2_-75 modified separators at a scan rate of 0.1 mV/s; Table S1: The ratio between the second plateau (II) and discharge capacity of the cells with various separators; Table S2: Thicknesses of coating in CeO_2_-25, CeO_2_-50, CeO_2_-75 and CeO_2_-100 modified separators.

## References

[1] Yao H., Yan K., Li W., Zheng G., Kong D., Seh Z.W., Narasimhan V.K., Liang Z., Cui Y. (2014). Improved lithium–sulfur batteries with a conductive coating on the separator to prevent the accumulation of inactive S-related species at the cathode–separator interface. Energy Environ. Sci..

[2] Ansari Y., Zhang S., Wen B., Fan F., Chiang Y.M. (2019). Stabilizing Li–S battery through multilayer encapsulation of sulfur. Adv. Energy Mater..

[3] Pei F., Lin L., Fu A., Mo S., Ou D., Fang X., Zheng N. (2018). A two-dimensional porous carbon-modified separator for high-energy-density Li-S batteries. Joule.

[4] Chung S.-H., Chang C.-H., Manthiram A. (2016). A core–shell electrode for dynamically and statically stable Li–S battery chemistry. Energy Environ. Sci..

[5] Cheng P., Guo P., Sun K., Zhao Y., Liu D., He D. (2021). CeO_2_ decorated graphene as separator modification material for capture and boost conversion of polysulfide in lithium-sulfur batteries. J. Membr. Sci..

[6] Xiao D., Lu C., Chen C., Yuan S. (2018). CeO_2_-webbed carbon nanotubes as a highly efficient sulfur host for lithium-sulfur batteries. Energy Storage Mater..

[7] Hong X.-J., Song C.-L., Yang Y., Tan H.-C., Li G.-H., Cai Y.-P., Wang H. (2019). Cerium based metal–organic frameworks as an efficient separator coating catalyzing the conversion of polysulfides for high performance lithium–sulfur batteries. ACS Nano.

[8] Gueon D., Yoon J., Hwang J.T., Moon J.H. (2020). Microdomain sulfur-impregnated CeO_2_-coated CNT particles for high-performance Li-S batteries. Chem. Eng. J..

[9] Ma L., Chen R., Zhu G., Hu Y., Wang Y., Chen T., Liu J., Jin Z. (2017). Cerium oxide nanocrystal embedded bimodal micromesoporous nitrogen-rich carbon nanospheres as effective sulfur host for lithium–sulfur batteries. ACS Nano.

[10] Wang L., Dong Z., Wang D., Zhang F., Jin J. (2013). Covalent bond glued sulfur nanosheet-based cathode integration for long-cycle-life Li–S batteries. Nano Lett..

[11] Hwa Y., Seo H.K., Yuk J.-m., Cairns E.J. (2017). Freeze-dried sulfur–graphene oxide–carbon nanotube nanocomposite for high sulfur-loading lithium/sulfur cells. Nano Lett..

[12] Parekh M.H., Rao H., Jokhakar D., Parikh V.P., Palanisamy M., Pol V.G. (2022). Polysulfide shuttle mitigation through a tailored separator for critical temperature energy-dense lithium–sulfur batteries. Sustain. Energy Fuels.

[13] Xue W., Yan Q.-B., Xu G., Suo L., Chen Y., Wang C., Wang C.-A., Li J. (2017). Double-oxide sulfur host for advanced lithium-sulfur batteries. Nano Energy.

[14] Li N., Xie Y., Peng S., Xiong X., Han K. (2020). Ultra-lightweight Ti_3_C_2_T_x_ MXene modified separator for Li–S batteries: Thickness regulation enabled polysulfide inhibition and lithium ion transportation. J. Energy Chem..

[15] Cui Z., Zu C., Zhou W., Manthiram A., Goodenough J.B. (2016). Mesoporous titanium nitride-enabled highly stable lithium-sulfur batteries. Adv. Mater..

[16] Xu H., Manthiram A. (2017). Hollow cobalt sulfide polyhedra-enabled long-life, high areal-capacity lithium-sulfur batteries. Nano Energy.

[17] Ye B., Feng C., Zhu G., Wang S., Fakhri A. (2020). Feather duster liked CeO_2_ as efficient adsorber host material for advanced lithium–sulfur batteries. J. Alloys Compd..

[18] Wang S., Gao F., Zhao Y., Liu N., Tan T., Wang X. (2018). Two-dimensional CeO_2_/RGO composite-modified separator for lithium/sulfur batteries. Nanoscale Res. Lett..

[19] Kim M., Lee J., Jeon Y., Piao Y. (2019). Phosphorus-doped graphene nanosheets anchored with cerium oxide nanocrystals as effective sulfur hosts for high performance lithium–sulfur batteries. Nanoscale.

[20] Li X., Zhang Y., Wang S., Liu Y., Ding Y., He G., Jiang X., Xiao W., Yu G. (2020). Scalable high-areal-capacity Li–S batteries enabled by sandwich-structured hierarchically porous membranes with intrinsic polysulfide adsorption. Nano Lett..

[21] Zhang H., Zhao W., Zou M., Wang Y., Chen Y., Xu L., Wu H., Cao A. (2018). 3D, mutually embedded MOF@ carbon nanotube hybrid networks for high-performance lithium-sulfur batteries. Adv. Energy Mater..

[22] Geng P., Wang L., Du M., Bai Y., Li W., Liu Y., Chen S., Braunstein P., Xu Q., Pang H. (2022). MIL-96-Al for Li–S Batteries: Shape or Size?. Adv. Mater..

[23] Razzaq A.A., Yuan X., Chen Y., Hu J., Mu Q., Ma Y., Zhao X., Miao L., Ahn J.-H., Peng Y. (2020). Anchoring MOF-derived CoS_2_ on sulfurized polyacrylonitrile nanofibers for high areal capacity lithium–sulfur batteries. J. Mater. Chem. A.

[24] Deng N., Wang L., Feng Y., Liu M., Li Q., Wang G., Zhang L., Kang W., Cheng B., Liu Y. (2020). Co-based and Cu-based MOFs modified separators to strengthen the kinetics of redox reaction and inhibit lithium-dendrite for long-life lithium-sulfur batteries. Chem. Eng. J..

[25] Jiang G., Zheng N., Chen X., Ding G., Li Y., Sun F., Li Y. (2019). In-situ decoration of MOF-derived carbon on nitrogen-doped ultrathin MXene nanosheets to multifunctionalize separators for stable Li-S batteries. Chem. Eng. J..

[26] Zhou Z., Li Y., Fang T., Zhao Y., Wang Q., Zhang J., Zhou Z. (2019). MOF-derived Co_3_O_4_ polyhedrons as efficient polysulfides barrier on polyimide separators for high temperature lithium–sulfur batteries. Nanomaterials.

[27] Elhussein E.A.A., Şahin S., Bayazit Ş.S. (2018). Preparation of CeO_2_ nanofibers derived from Ce-BTC metal-organic frameworks and its application on pesticide adsorption. J. Mol. Liq..

[28] Chen X., Yu E., Cai S., Jia H., Chen J., Liang P. (2018). In situ pyrolysis of Ce-MOF to prepare CeO_2_ catalyst with obviously improved catalytic performance for toluene combustion. Chem. Eng. J..

[29] Guo Y., Yu Q., Fang H., Wang H., Han J., Ge Q., Zhu X. (2020). Ce–UiO-66 Derived CeO_2_ Octahedron Catalysts for Efficient Ketonization of Propionic Acid. Ind. Eng. Chem. Res..

[30] Zhang X., Hou F., Yang Y., Wang Y., Liu N., Chen D., Yang Y. (2017). A facile synthesis for cauliflower like CeO_2_ catalysts from Ce-BTC precursor and their catalytic performance for CO oxidation. Appl. Surf. Sci..

[31] Wang R., Luo C., Wang T., Zhou G., Deng Y., He Y., Zhang Q., Kang F., Lv W., Yang Q.H. (2020). Bidirectional catalysts for liquid–solid redox conversion in lithium–sulfur batteries. Adv. Mater..

[32] Zhang N., Yang Y., Feng X., Yu S.-H., Seok J., Muller D.A., Abruña H.D. (2019). Sulfur encapsulation by MOF-derived CoS_2_ embedded in carbon hosts for high-performance Li–S batteries. J. Mater. Chem. A.

[33] Abdelkader A.A., Rodene D.D., Norouzi N., Alzharani A., Weeraratne K.S., Gupta R.B., El-Kaderi H.M. (2020). Multifunctional Electrocatalytic Cathodes Derived from Metal–Organic Frameworks for Advanced Lithium-Sulfur Batteries. Chem.–A Eur. J..

[34] Li H., Chen C., Yan Y., Yan T., Cheng C., Sun D., Zhang L. (2021). Utilizing the Built-in Electric Field of p–n Junctions to Spatially Propel the Stepwise Polysulfide Conversion in Lithium–Sulfur Batteries. Adv. Mater..

[35] Xu J., Zhang W., Chen Y., Fan H., Su D., Wang G. (2018). MOF-derived porous N–Co_3_O_4_@N–C nanododecahedra wrapped with reduced graphene oxide as a high capacity cathode for lithium–sulfur batteries. J. Mater. Chem. A.

[36] Li W., Qian J., Zhao T., Ye Y., Xing Y., Huang Y., Wei L., Zhang N., Chen N., Li L. (2019). Boosting High-Rate Li–S Batteries by an MOF-Derived Catalytic Electrode with a Layer-by-Layer Structure. Adv. Sci..

[37] Sun T., Zhao X., Li B., Shu H., Luo L., Xia W., Chen M., Zeng P., Yang X., Gao P. (2021). NiMoO_4_ Nanosheets Anchored on N–S Doped Carbon Clothes with Hierarchical Structure as a Bidirectional Catalyst toward Accelerating Polysulfides Conversion for Li–S Battery. Adv. Funct. Mater..

[38] Jiao L., Zhang C., Geng C., Wu S., Li H., Lv W., Tao Y., Chen Z., Zhou G., Li J. (2019). Capture and Catalytic Conversion of Polysulfides by In Situ Built TiO_2_-MXene Heterostructures for Lithium–Sulfur Batteries. Adv. Energy Mater..

[39] Zhou G., Tian H., Jin Y., Tao X., Liu B., Zhang R., Seh Z.W., Zhuo D., Liu Y., Sun J. (2017). Catalytic oxidation of Li_2_S on the surface of metal sulfides for Li–S batteries. Proc. Natl. Acad. Sci. USA.

[40] Kresse G., Furthmüller J. (1996). Efficient iterative schemes for ab initio total-energy calculations using a plane-wave basis set. Phys. Rev. B.

[41] Kresse G., Furthmüller J. (1996). Efficiency of ab-initio total energy calculations for metals and semiconductors using a plane-wave basis set. Comput. Mater. Sci..

[42] Monkhorst H.J., Pack J.D. (1976). Special points for Brillouin-zone integrations. Phys. Rev. B.

[43] Grimme S., Ehrlich S., Goerigk L. (2011). Effect of the damping function in dispersion corrected density functional theory. J. Comput. Chem..

[44] Momma K., Izumi F. (2008). VESTA: A three-dimensional visualization system for electronic and structural analysis. J. Appl. Crystallogr..

