# Supplementary material for: MOF-Derived CeO2 Nanorod as a Separator Coating Enabling Enhanced Performance for Lithium–Sulfur Batteries
Source: Molecules. 2024 Apr 18;29(8):1852. doi: 10.3390/molecules29081852 (PMC11054290; doi:10.3390/molecules29081852)
Supplement: Supplementary file 1 [file molecules-29-01852-s001.zip › molecules-2943540-supplementary.pdf]

Supporting information

MOF-Derived CeO_2 Nanorod as a Separator Coating Enabling Enhanced Performance for Lithium–Sulfur Batteries

Hao Xiao [1,2,3,4,5], Jian Qin [1,2,3,4,5], Haodong Wang [2,3,4,5], Xiaoxu Lai [2,3,4,5], Pei Shi [2,3,4,5], Chi Chen [2,3,4,5,*] and Dan Sun [2,3,4,5,*]

[1] College of Chemistry, Fuzhou University, Fuzhou 350116, China; xmxiaohao@fjirsm.ac.cn (H.X.); xmqinjian@fjirsm.ac.cn (J.Q.)
[2] CAS Key Laboratory of Design and Assembly of Functional Nanostructures, and Fujian Provincial Key Laboratory of Nanomaterials, Fujian Institute of Research on the Structure of Matter, Chinese Academy of Sciences, Fuzhou 350002, China; whd19855695910@163.com (H.W.); xmlaixiaoxu@fjirsm.ac.cn (X.L.); 348936@whut.edu.cn (P.S.)
[3] Xiamen Institute of Rare Earth Materials, Haixi Institutes, Chinese Academy of Sciences, Xiamen 361021, China
[4] Xiamen Key Laboratory of Rare Earth Photoelectric Functional Materials, Xiamen 361021, China
[5] Fujian College, University of Chinese Academy Sciences, Fuzhou 350002, China
* Correspondence: xmchenchi@fjirsm.ac.cn (C.C.); xmsundan@fjirsm.ac.cn (D.S.)

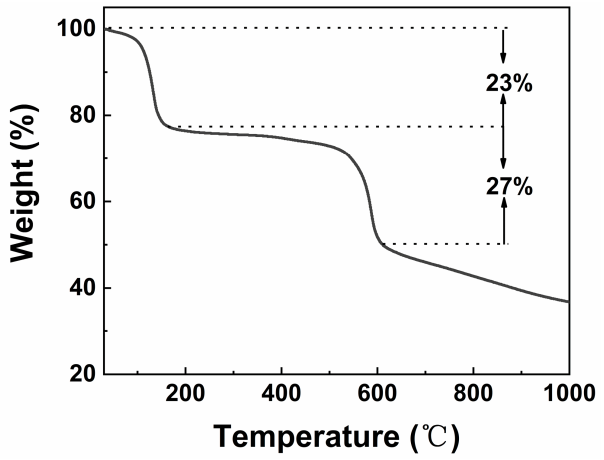

Figure S1. Thermogravimetric analysis curve of Ce–MOF to determine the calcination temperature for the synthesis of CeO_2.

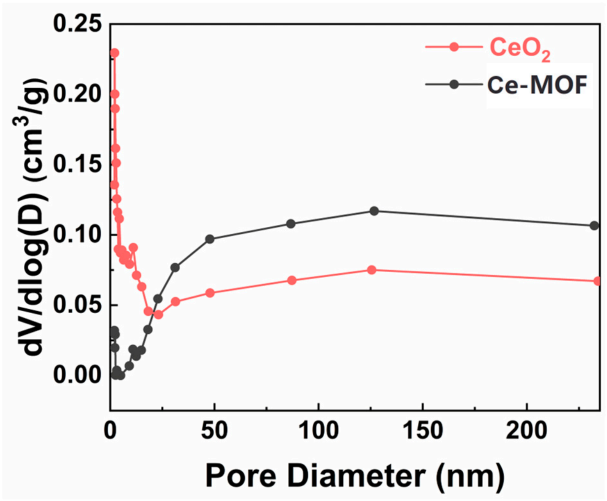

Figure S2. Pore size distributions of Ce–MOF and CeO_2, respectively.

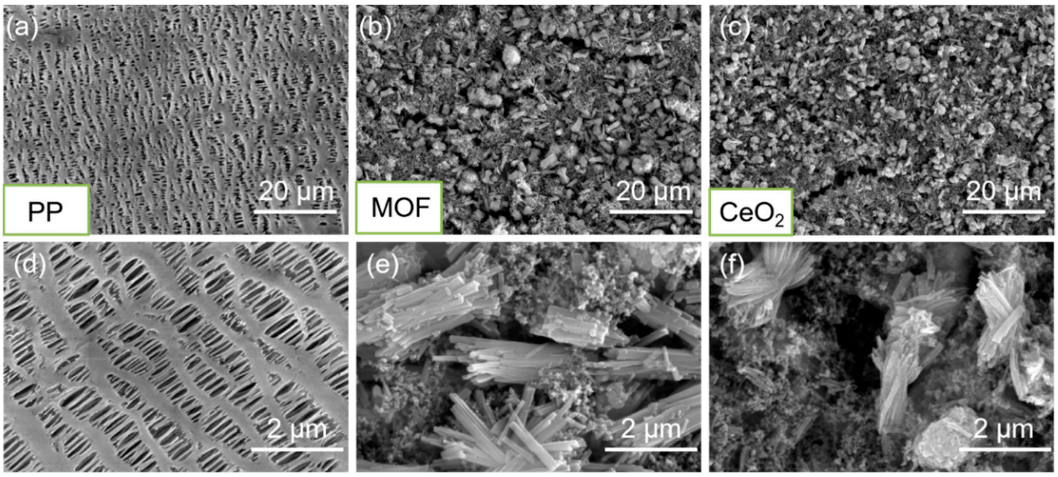

Figure S3. SEM images of (a, d) pristine polypropylene (PP) separator, (b, e) Ce–MOF modified separator and (c, f) CeO_2 modified separator.

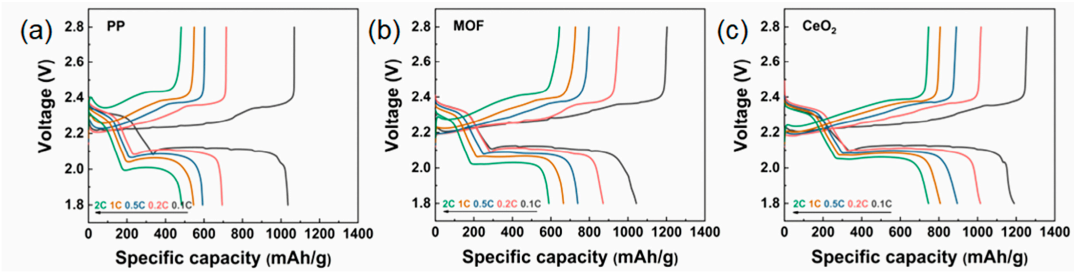

Figure S4. Charge–discharge profiles of Li–S batteries with (a) PP separator, (b) Ce–MOF, and (c) CeO_2 modified separators at different current densities of 0.1, 0.2, 0.5, 1, 2 C.

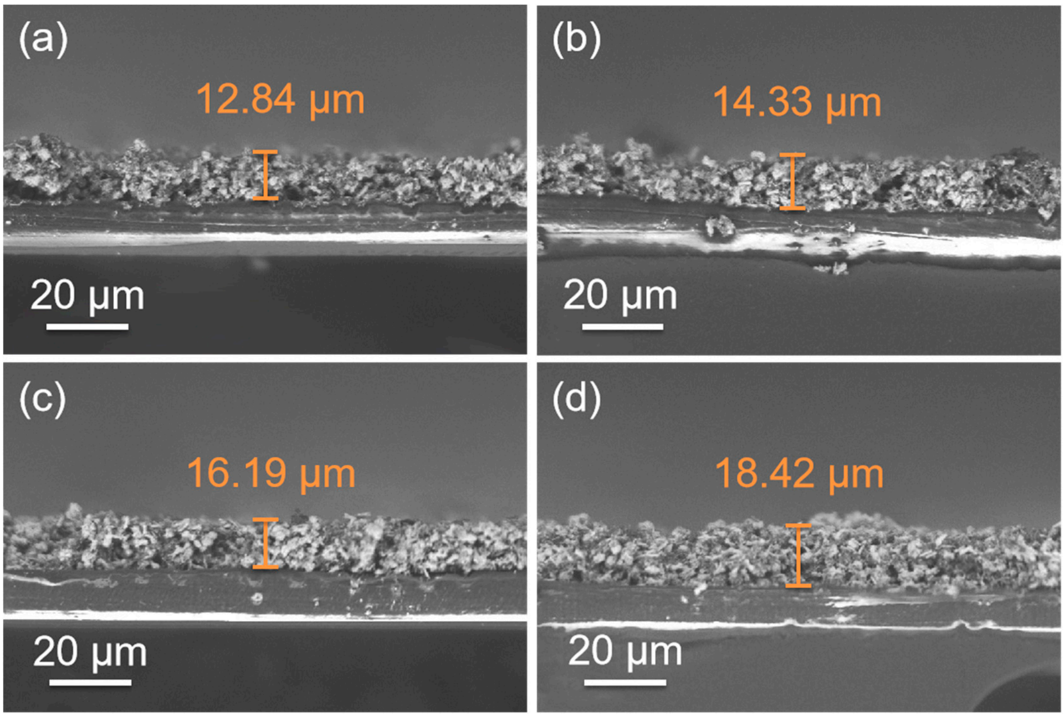

Figure S5. Cross–sectional SEM images of (a) CeO_2–25, (b) CeO_2–50, (c) CeO_2–75 and (d) CeO_2–100 modified separators.

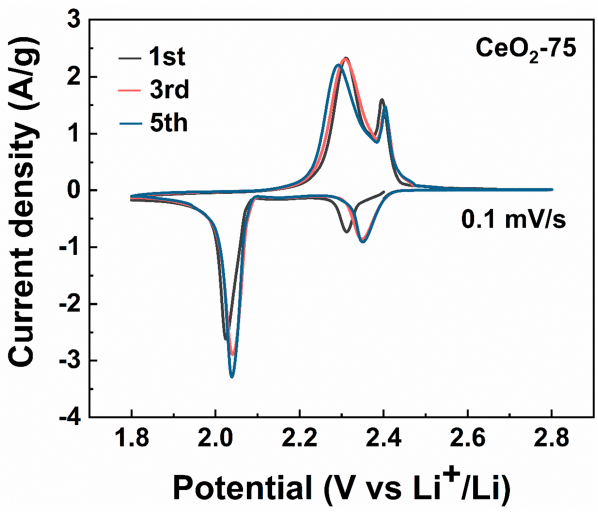

Figure S6. The first, third and fifth cycled CV curves of Li-S batteries with CeO_2–75 modified separators at a scan rate of 0.1 mV/s.

Table S1. The ratio between the second plateau (II) and discharge capacity of the cells with various separators.

II (%)	Current density				
	0.1 C	0.2 C	0.5 C	1 C	2 C
PP	68.10 %	66.00 %	59.20 %	62.50 %	60.50 %
MOF	72.20 %	67.00 %	66.00 %	67.80 %	67.60 %
CeO_2	71.20 %	67.00 %	67.00 %	65.70 %	64.40 %

Table S2. Thicknesses of coating in CeO_2–25, CeO_2–50, CeO_2–75 and CeO_2–100 modified separators.

Sample	Thickness (μm)
CeO_2–25	12.84
CeO_2–50	14.33

CeO_2–75	16.19
CeO_2–100	18.42
